# Blood-Derived Metabolic Signatures as Biomarkers of Injury Severity in Traumatic Brain Injury: A Pilot Study

**DOI:** 10.3390/metabo14020105

**Published:** 2024-02-02

**Authors:** Elani A. Bykowski, Jamie N. Petersson, Sean P. Dukelow, Chester Ho, Chantel T. Debert, Tony Montina, Gerlinde A. S. Metz

**Affiliations:** 1 Canadian Centre for Behavioural Neuroscience, Department of Neuroscience, University of Lethbridge, Lethbridge, AB T1K 3M4, Canada; elani@ualberta.ca (E.A.B.); jamie.petersson@uleth.ca (J.N.P.); 2 Southern Alberta Genome Sciences Centre, University of Lethbridge, Lethbridge, AB T1K 3M4, Canada; 3 Department of Chemistry and Biochemistry, University of Lethbridge, Lethbridge, AB T1K 3M4, Canada; 4 Department of Clinical Neurosciences, Cumming School of Medicine, University of Calgary, Calgary, AB T2N 1N4, Canada; sean.dukelow@albertahealthservices.ca; 5 Hotchkiss Brain Institute, University of Calgary, Calgary, AB T2N 1N4, Canada; 6 Division of Physical Medicine and Rehabilitation, University of Alberta, Edmonton, AB T6G 2R7, Canada; chester.ho@albertahealthservices.ca

**Keywords:** metabolomics, blood, traumatic brain injury, concussion, nuclear magnetic resonance (NMR) spectroscopy, symptom burden, severity, recovery, precision medicine, rehabilitation, biological pathways

## Abstract

Metabolomic biomarkers hold promise in aiding the diagnosis and prognostication of traumatic brain injury. In Canada, over 165,000 individuals annually suffer from a traumatic brain injury (TBI), making it one of the most prevalent neurological conditions. In this pilot investigation, we examined blood-derived biomarkers as proxy measures that can provide an objective approach to TBI diagnosis and monitoring. Using a ^1^H nuclear magnetic resonance (NMR)-based quantitative metabolic profiling approach, this study determined whether (1) blood-derived metabolites change during recovery in male participants with mild to severe TBI; (2) biological pathway analysis reflects mechanisms that mediate neural damage/repair throughout TBI recovery; and (3) changes in metabolites correlate to initial injury severity. Eight male participants with mild to severe TBI (with intracranial lesions) provided morning blood samples within 1–4 days and again 6 months post-TBI. Following NMR analysis, the samples were subjected to multivariate statistical and machine learning-based analyses. Statistical modelling displayed metabolic changes during recovery through group separation, and eight significant metabolic pathways were affected by TBI. Metabolic changes were correlated to injury severity. L-alanine (R= −0.63, *p* < 0.01) displayed a negative relationship with the Glasgow Coma Scale. This study provides pilot data to support the feasibility of using blood-derived metabolites to better understand changes in biochemistry following TBI.

## 1. Introduction

As the leading cause of death and disability worldwide, traumatic brain injury (TBI) is the “silent epidemic” that afflicts millions of individuals annually [1]. TBI pathology is characterized by two distinct phases. At first, TBI results in primary injury, or the sequelae resulting from mechanical forces at the time of impact, leading to bruising, bleeding, and tearing of fibers within the cranial tissue [2]. The delayed secondary injury phase provides opportunities for therapeutic intervention. This phase is characterized by disturbances in brain metabolism, which can lead to pathophysiological changes, such as neurodegeneration, and persistent physical, cognitive, and somatic symptoms [3]. As a prevalent and rising health issue, TBI would benefit from more objective measures for its diagnosis and treatment. An objective and quantitative blood biomarker could be an important adjunctive tool for early diagnosis and could provide a window into the pathophysiological processes in the body. It would also offer the ability to assist in decision-making about the mode of intervention that would most effectively optimize the personal potential for recovery. To the authors’ knowledge, the current investigation represents the first attempt to identify NMR-based serum metabolite markers that could serve as indicators of diagnosis and the extent of recovery in TBI patients using a longitudinal model and human participants.

There have been many previous efforts to identify biomarkers with diagnostic and prognostic potential, including S100B and glial fibrillary acidic protein (GFAP), which have led to their use in a clinical setting for TBI diagnosis [4,5]. A metabolite biomarker in addition to these protein markers may further reinforce diagnostic testing accuracy. In an effort to expand the repository for potential biomarkers, our team has shown that metabolites in urine samples collected from athletes with concussion resulted in significantly distinct metabolic profiles at baseline and post-injury [6]. Furthermore, urine and serum sample analysis provided robust metabolic biomarkers for spinal cord injury (SCI) and TBI using ^1^H nuclear magnetic resonance (NMR) spectroscopy [7,8,9]. ^1^H NMR spectroscopy is especially amenable to detecting metabolic changes in the blood, as it can detect 49 compounds, with 20 of these being unique to NMR [10]. Thus, ^1^H NMR spectroscopy is instrumental to the rapidly growing field of metabolomics, whereby the metabolic fingerprint of an individual is captured using endogenous small molecules within biological fluids [11]. In addition, metabolomics studies have successfully identified biomarkers for neurological disorders, including Alzheimer’s disease [12], multiple sclerosis [13], and Parkinson’s disease [14].

The aim of the present study was to establish potential biomarker profiles for traumatic brain injury that accurately reflect clinical symptom severity. In this prospective cohort study, blood samples from male participants with mild to severe TBI were examined to address the following objectives: (1) to determine metabolic differences in the initial (within 1–4 days post-injury) and 6 months post-injury metabolomics profiles; (2) based on the list of significant metabolites, to reveal the underlying biochemical pathways; and (3) to examine how these changes correlate to the severity of the injury.

## 2. Materials and Methods

### 2.1. Participant Characteristics and Study Design

This exploratory pilot prospective cohort study was nested in a larger study called Understanding Neurological Recovery: The Role of Resting-state fMRI, Biomarkers, and Robotics After TBI, Stroke, and SCI (UCAN Study), supported by the Hotchkiss Brain Institute at the University of Calgary. The UCAN Study followed participants with TBI, SCI, and stroke throughout their recovery trajectory from one week to 6 months post-injury. Twelve participants with TBI were recruited through the Foothills Medical Centre, Calgary. Patient demographics and injury characteristics were collected after obtaining consent. Out of the twelve, eight male participants (Table 1; average age 45 +/− 18.4 years) provided fasting morning (between 6 am and 9 am) blood samples at two different time points: initially after TBI and again at approximately 6 months post-injury. The initial sample collection was completed within 1–4 days following TBI (median = 2.5, interquartile range = 1.25) and the 6-month time-point samples were collected within 184–312 days following injury (median = 200, interquartile range = 24.5). Pairing the samples for this within-subject control study minimizes the confounding factors, thereby increasing the validity of the analysis to attribute changes in the metabolic profiles throughout recovery. Whole blood samples were immediately centrifuged and spun down to isolate the serum, and the serum samples were frozen, transported to the University of Lethbridge, and stored in a −80 degrees Celsius freezer until NMR sample preparation and data acquisition. This study was approved by the University of Calgary’s conjoint ethics board (ethics ID: REB14-1017).

### 2.2. Clinical Assessment

The Glasgow Coma Scale (GCS) was used to rate each participant’s initial TBI severity and was determined within 24 h following injury (*n* = 2 severe, *n* = 3 moderate, *n* = 3 mild). The GCS measured eye opening on a scale of 1–4, the verbal response was scored from 1–5, and motor responses were recorded with scores measured from 1–6, with higher scores marking greater function [15]. The scores for each measure were summed to obtain a final score that indicated severity within the following ranges: severe (GCS less than 8), moderate (GCS 8–12), and mild (GCS 13–15).

The Montreal Cognitive Assessment (MoCA) and the Functional Independence Measure (FIM) were administered to determine both the injury severity and recovery [16,17]. The MoCA was assessed within 4–70 days after TBI (median = 30, interquartile range = 20.5) and at 6 months (184–312 days) following injury (median = 200, interquartile range = 24.5). The MoCA assessed short-term memory, visuospatial abilities, executive functions, and language. A MoCA score below 26 indicates an impairment, while a score greater than or equal to 26 is considered normal. The FIM is a global assessment of physical, social, and psychological function. This assessment evaluates self-care, continence, mobility, transfers, communication, and cognition. Each of the 18 items was rated on a scale from 1–7, with a score of 1 indicating total dependence and a score of 7 indicating complete independence. The total score is a value between 18 and 126 and suggests the level of function.

### 2.3. NMR Sample Preparation, Data Acquisition, and Processing

All the serum samples were processed for metabolite extraction in a containment level 2 (CL2) laboratory at the University of Lethbridge. The samples were slowly thawed on ice, and 200 µL of each serum sample and 300 µL of buffer (4:1 ratio of 0.625 M K_2_HPO_4_:KH_2_PO_4_ in dH_2_O–pH 7.4, 3.75 mM NaN_3_, and 0.375 M KF) were pipetted into a 0.5 mL 3 kDa centrifuge filter and centrifuged at 14,000× *g* for 30 min at 4 °C. Subsequently, 380 µL of the filtrate (which contained the small molecule metabolites), 100 µL of buffer, and 120 µL of 0.02709% weight/volume D2O with trimethylsilyl propanoic acid (TSP) were pipetted into a new microfuge tube and centrifuged at 12,000 rpm for 5 min at 4 °C. A total of 550 µL of the supernatant was then transferred to an NMR tube to be loaded into the spectrometer. The TSP in the D2O served as a chemical shift reference for the 1H NMR spectroscopy, and all the centrifuge filters were washed 10 times with deionized water immediately prior to use in order to remove the glycerol preservative from the filters.

A 700 MHz Bruker Avance III HD NMR spectrometer (Bruker Ltd., Billerica, MA, USA) and a room-temperature TBO-Z probe were used to acquire the NMR data. Three-dimensional and one-dimensional shimming experiments were conducted prior to NMR data acquisition on the serum samples to correct for any inhomogeneities in the static magnetic field. In addition, the 90-degree pulse width was calibrated for each sample prior to data acquisition. The data were acquired using a one-dimensional 1H Nuclear Overhauser Effect Spectroscopy experiment with gradient-based water suppression (‘noesygppr1d’ pulse sequence) with a mixing time of 10 ms. The experiment was conducted using 128 k data points, an acquisition time of 4.56 s, a recycle delay of 1 s, a constant receiver gain across all experiments, 128 scans, and a sample temperature of 22 degrees Celsius. The data were processed in Bruker TopSpin software (v. 3.5 pl7) using zero filling to 256 k points, line broadening to 0.3 Hz, and automatic phase and baseline correction. The processed NMR spectra were then exported as ASCII files.

### 2.4. Statistical Analysis

The ASCII files for each processed NMR spectra were imported into MATLAB (R2015b, MathWorks, Natick, MA, USA) where they underwent dynamic adaptive binning [18], followed by manual inspection and correction of the bins. In total, 246 bins were created for this analysis, and the region corresponding to the water signal was excluded from the final spectral bins. In addition, metabolite peaks near the water signal were only included if they were baseline resolved from the water peak for all the spectra (i.e., peaks on the “tails” of the suppressed water peak were not included). This exclusion minimizes or eliminates any effects that the water suppression method has on the quantification of metabolites. The bins corresponding to each NMR spectra were normalized to the total metabolome (sum or all bins for each spectrum), excluding the water region, Pareto-scaled, and log-transformed [19,20,21,22]. The metabolite regulation reported (percent regulation) is based on the changes in the normalized concentration of each metabolite (bin) using the following formula:

$$\frac{6\;month\;spost\;injury-initial}{\left(\frac{6\;month\;spost\;injury+initial}{2}\right)}\times 100$$

Multivariate statistical analysis provided insights into whether metabolite signatures could be used to discriminate between the initial and 6 months post-injury serum samples. The metabolites of the strongest importance were classified based on Variable Importance Analysis based on random Variable Combination (VIAVC) [23]. This MATLAB-based machine-learning algorithm enabled the identification of significant metabolites based on the Receiver Operator Characteristic (ROC) test and the subsequent Area-Under-the-Curve (AUC) analysis [24]. It also employed a binary matrix resampling method, which is a more robust method for randomly sampling the data, and all the multivariate supervised models were double ten-fold cross-validated and underwent permutation testing using 2000 permutations [25]. Univariate statistical tests were also conducted using either a paired *t*-test or a paired Wilcoxon–Mann–Whitney test in the case of parametric or non-parametric data, respectively. To determine the parametricity of each bin, a Shapiro–Wilk test was used [26].

To visualize supervised between-group and within-group separation, an orthogonal projection to latent structures discriminant analysis (OPLS-DA) was performed. The advantage of using OPLS-DA is that the model is rotated where class separation or between-class, correlated variation is found in the first predictive component and within-class, uncorrelated variation is seen in the orthogonal component [22]. Additionally, Principal Components Analysis (PCA) was conducted, which illustrated the degree of unsupervised separation. The OPLS-DA and PCA modelling, as well as the pathway topology analysis outlined below, were carried out using MetaboAnalystR version 2.0.4 running inside R version 3.5.3 [27].

Pearson R correlations were computed between the concentrations of blood-derived metabolites and the Glasgow Coma Scale (GCS) scores. The 6 months post-injury normalized concentrations were subtracted from the initial normalized concentrations to generate the change in the metabolite concentration used in the correlations. A Bonferroni corrected *p*-value, obtained by dividing α < 0.05 by the number of VIAVC F-ranked bins tested for each analysis (α < 0.005), was used to obtain a more rigorous set of clinically relevant metabolites [26]. The metabolites corresponding to significantly altered bins were identified using a combination of resources: Chenomx 8.2 NMR Suite (Chenomx Inc., Edmonton, Alberta, Canada), the Human Metabolome Database (HMBD) [28], and the Human Serum Metabolome [10] containing a list of chemical classes of blood-based metabolites. Pathway topology analysis was conducted using a hypergeometric test for over-representation analysis and relative-betweenness centrality for topology analysis. This analysis utilized the complete list of significant metabolites, the Kyoto Encyclopedia of Genes and Genomes (KEGG) database, and the HMBD libraries [28,29] to provide the metabolic pathways that have been potentially altered following TBI.

## 3. Results

### 3.1. Clinical Demographics

The number of participants with severe (*n* = 2), moderate (*n* = 3), and mild GCS (*n* = 3) are summarized in Table 1. The majority of TBI participants displayed clinical improvement in the MoCA and FIM scores after 6 months, with an average improvement of 2.375 ± 2.504 and 4.5 ± 5.127, respectively.

### 3.2. Metabolomic Profiles Significantly Change over Time following TBI

Of the 246 bins created for this analysis, 41 and 3 bins were determined to be significantly altered by univariate (Paired *t*-test or Wilcoxon–Mann–Whitney test) or VIAVC best subset, respectively. The metabolites corresponding to these bins are provided in Table 2 and are ranked in order of significance according to the *p*-value obtained from the paired *t*-test/Wilcoxon–Mann–Whitney analysis. The metabolites that were significant by VIAVC best subset include 2-hydroxybutyrate and L-alanine. Among the top metabolites significant by paired *t*-test were L-phenylalanine, 1,9-dimethyluric acid, phosphonoacetate, p-cresol, and glycine.

**Figure 1:**
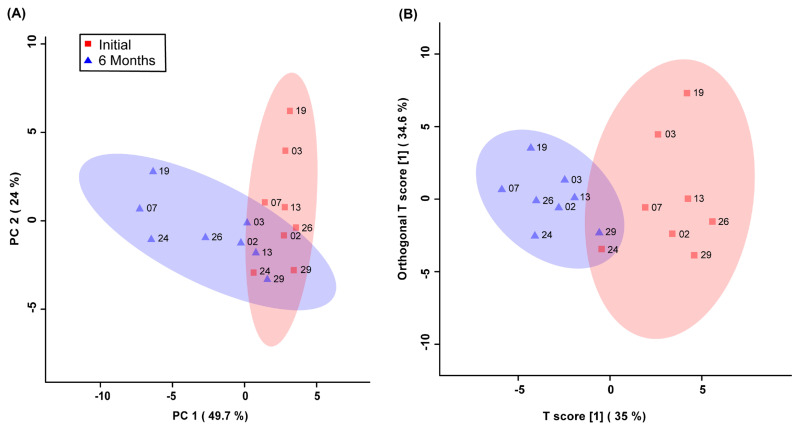
Principal Components Analysis (PCA; (**A**)) and Orthogonal Projections to Latent Structures Discriminant Analysis (OPLS-DA; (**B**)) scores plots. This analysis was carried out using a list of blood-derived metabolites found to be statistically significant by paired *t*-test and VIAVC best subset testing. Red color and squares indicate participants at the initial time-point, and blue color and triangles indicate participants at the 6-month time-point. The participant code (as seen in Table 1) is labelled beside their corresponding square or triangle. The 95% confidence interval is indicated by the shaded ellipses. In the case of the PCA scores plot, the x-axis and y-axis show the data variance explained by principal components 1 and 2, respectively. In the case of the OPLS-DA scores plot the x-axis and y-axis show the predictive (between-group) and orthogonal (within-group) variation, respectively. The following are the cross-validation and permutation measures for the OPLS-DA figure: R^2^Y = 0.794 (*p* < 0.01), Q^2^ = 0.607 (*p* < 0.01).

**Figure 2:**
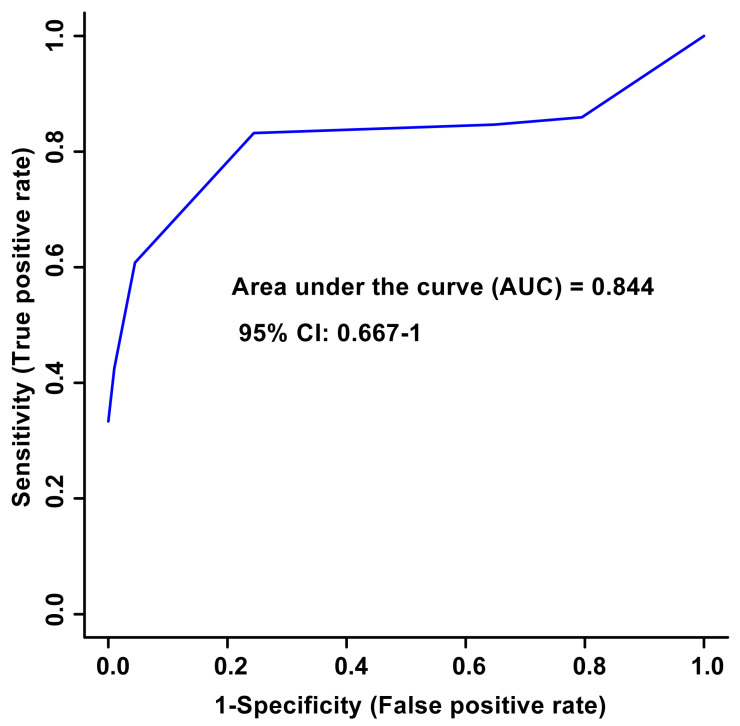
Receiver Operator Characteristic (ROC) curve and the corresponding area under the curve (AUC) and confidence interval are indicated in the figure. This figure was generated using the significantly altered VIAVC best subset metabolites, which corresponds to 3 bins. The predictive accuracy is 86.3% when all the bins from the best subset are considered.

Unsupervised PCA multivariate modelling utilizing all the bins showed a complete overlap of the groups when comparing the metabolome across the two time points, while supervised OPLS-DA modelling utilizing all the bins did not pass permutation or cross-validation testing. Subsequently, the subset of bins determined to be significantly altered by univariate or multivariate testing was then utilized to carry out both unsupervised and supervised multivariate modelling. The PCA scores plot demonstrated a partial degree of unsupervised group separation (Figure 1A), while the supervised OPLS-DA scores plot illustrated significant group separation between the initial injury and 6 months post-injury samples (R^2^Y = 0.794, *p* < 0.01; Q^2^ = 0.607, *p* < 0.01, Figure 1B). This supervised model indicated a change in the metabolic profiles following TBI over the course of the recovery process. In addition, ROC curves were generated to determine the predictive accuracy of the model, and an area under the curve equal to 0.844 was achieved with a 95% confidence interval of 0.667-1 (Figure 2).

**Figure 3:**
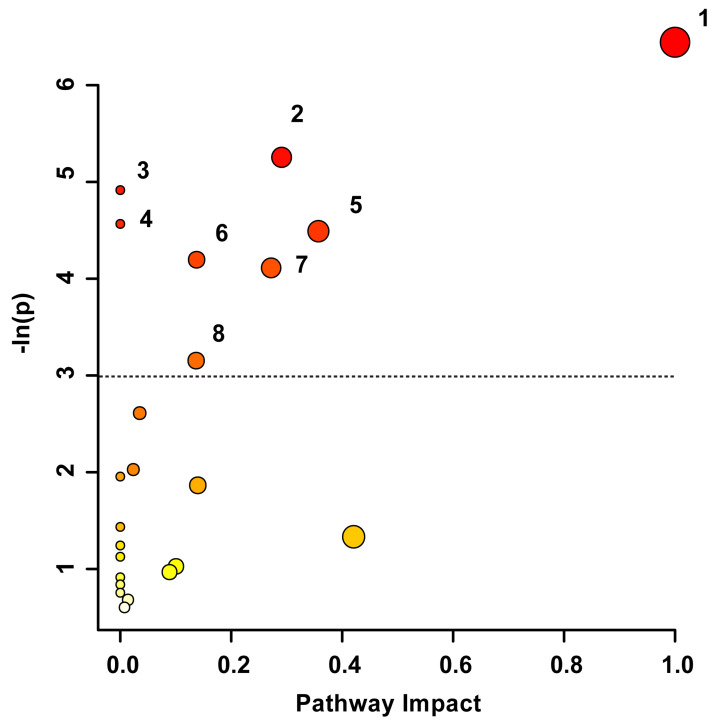
Metabolic Pathway Topology Analysis. A higher value on the y-axis and a darker circle color indicate a lower *p*-value for the pathway. A higher value on the x-axis and larger circle size indicates greater pathway impact, which is a measure of how affected each pathway is by the metabolites identified as significantly altered. Only pathways with a *p*-value less than 0.05 are labelled, with the cut-off indicated by the dotted line. This analysis was carried out using the list of metabolites that were identified to be significantly altered by the paired *t*-test/Mann–Whitney test and the VIAVC best subset. The numbering corresponds to significantly altered pathways: 1. Phenylalanine, tyrosine, and tryptophan biosynthesis (*p* = 0.0016), 2. Pyruvate metabolism (*p* = 0.005), 3. Aminoacyl-tRNA biosynthesis (*p* = 0.007), 4. Alanine, aspartate, and glutamate metabolism (*p* = 0.01), 5. Phenylalanine metabolism (*p* = 0.011), 6. Glyoxylate and dicarboxylate metabolism (*p* = 0.015), 7. Glycine, serine, and threonine metabolism (*p* = 0.016), and 8. Citrate Cycle/TCA cycle (*p* = 0.043).

Pathway topology analysis was used to uncover the underlying biochemical pathways potentially altered due to TBI severity and recovery. The pathway analysis in Figure 3 illustrates the potential pathway impact based on changes to the participants’ blood-derived metabolic profiles, presented in increasing order of impact. The metabolic pathways significantly affected were phenylalanine, tyrosine, and tryptophan biosynthesis (*p* < 0.01), pyruvate metabolism (*p* < 0.01), aminoacyl-tRNA biosynthesis (*p* < 0.01), alanine, aspartate, and glutamate metabolism (*p* = 0.01), phenylalanine metabolism (*p* < 0.05), glyoxylate and dicarboxylate metabolism (*p* < 0.05), glycine, serine, and threonine metabolism (*p* < 0.05), and the citrate cycle (*p* < 0.05). The pathway analysis was based on bins significant by the VIAVC best subset and the paired *t*-test/or the Wilcoxon–Mann–Whitney test.

### 3.3. Metabolomic Signatures Correlate with Injury Severity

Pearson R correlation tests were performed to compare the change in the concentration of each VIAVC F-ranked metabolite (6 months post-injury concentration–initial concentration) to the GCS, FIM, and MoCA scores. There were ten metabolites that achieved significance according to the VIAVC F-ranked test. Of the ten metabolites tested, only one metabolite, L-Alanine (R = −0.63, *p* < 0.01), had a negative correlation to injury severity using GCS but was not below the Bonferroni corrected threshold (α < 0.005). There were no significant correlations observed between the metabolite concentrations and both the FIM and MoCA scores.

## 4. Discussion

### 4.1. General Discussion

The present study revealed specific blood-derived metabolites that significantly change during recovery following TBI. The metabolic changes were potentially associated with eight biochemical pathways: phenylalanine, tyrosine, and tryptophan biosynthesis; pyruvate metabolism; aminoacyl-tRNA biosynthesis; alanine, aspartate, and glutamate metabolism; phenylalanine metabolism; glyoxylate and dicarboxylate metabolism; glycine, serine, and threonine metabolism; and the citrate cycle. When correlating the changes in the metabolites throughout recovery, L-alanine significantly negatively correlated with injury severity using the GCS. These findings provide preliminary evidence that a metabolomics approach of blood samples combined with machine learning analysis has the potential to provide metabolic profiles associated with injury severity using the GCS.

As shown in Table 2, several metabolites showed changes in concentration when comparing the initial and 6 months post-injury samples. L-alanine, 1,3,7-trimethyluric acid, and 2-hydroxybutyrate were significant via VIAVC best subset. L-alanine is a non-essential amino acid that is discussed below because it was the single metabolite significantly correlated to GCS scores. 1,3,7-trimethyluric acid is a breakdown product of purines that may be neuroprotective [30]. 2-hydroxybutyrate is a ketone body, which is an alternate energy source in states of increased energetic demand, such as TBI. Thus, ketone bodies have been posited as a prospective therapeutic intervention in TBI [31].

Biochemical pathways were derived based on metabolites significant by VIAVC best subset and the paired *t*-test/Wilcoxon–Mann–Whitney test, of which phenylalanine, tyrosine, and tryptophan biosynthesis was the most significantly altered. The metabolites phenylalanine and tyrosine implicated in this pathway indicated disruptions to neurotransmitter signaling, as these amino acids are known precursors to catecholamine neurotransmitters, including dopamine and epinephrine. The large neutral amino acid transport (LNAA) system found at the blood–brain barrier (BBB) is the gateway for the uptake of these amino acids into the brain [32]. A TBI leads to disruption of the vessels within the BBB which can lead to ischemia in the surrounding areas [33]. Thus, it is plausible that BBB disruption and the ensuing changes in amino acid levels precipitate abnormalities in neurotransmitter production.

Pathway analysis also indicated that pyruvate metabolism was potentially altered, which is in agreement with a parallel study examining blood-derived biomarkers amongst male SCI participants, where it was found that pyruvate metabolism was the most significantly altered pathway [9]. Pyruvate is generated from the metabolism of glucose, and the lactate/pyruvate ratio is a clinically informative measure indicative of cerebral metabolic state [34]. An increase in this metric is a known indicator of TBI, signaling a switch from aerobic to anaerobic respiration, and can predict the outcomes [35]. This overlapping characteristic of SCI and TBI warrants further investigation as a potential clinical feature for central nervous system trauma in general.

Aminoacyl tRNA biosynthesis was potentially altered following TBI and plays a key role in protein biosynthesis, as aminoacyl tRNA synthetases are programmed in the genetic code throughout the cells of the body. tRNAs undergo modifications in response to cellular stress to regulate protein synthesis [36]. These modifications may involve the cleavage of tRNAs to generate fragments derived from them. Recently, several tRNA-derived fragments have been identified as biomarkers for brain injury diagnosis [37,38] and impaired outcomes following TBI [39]. Because the comprehension of the involvement of tRNAs in brain injury is still in the nascent stages, more research is imperative to deduce the role of aminoacyl tRNA and its synthesis in TBI.

Another pathway found to be potentially affected post-TBI was alanine, aspartate, and glutamate metabolism. In our parallel study examining blood-derived biomarkers amongst SCI subjects, this pathway also presented as potentially altered, as evidenced by pathway analysis [9]. It is known that the excitatory amino acids aspartate and glutamate are upregulated following insult to the spinal cord [40,41], and this may also be extrapolated to include TBI. Alanine is an inhibitory amino acid released in response to ischemia, oxidative stress, and free radical formation, as evidenced in the hippocampus [42]. Thus, we argue that, similar to SCI, TBI may induce changes along this physiological pathway, but more studies are needed to develop a thorough understanding.

Phenylalanine metabolism is implicated in neurotransmitter production and brain signaling, and the repeated significance of this pathway in the analysis strongly suggests that the disruption of neurotransmitters is a pathological process in the wake of TBI. Phenylalanine is a precursor to tyrosine [43], which gives rise to the neurotransmitters dopamine, epinephrine, and norepinephrine. Previous studies have also indicated that the levels of these neurotransmitters are associated with the severity of injury [44].

The present study revealed that the tricarboxylic acid (TCA) cycle and glyoxylate and dicarboxylate metabolism were both potentially altered following TBI. A parallel study conducted by our group found the metabolism of glyoxylate and dicarboxylate to be affected based on blood-derived metabolites amongst SCI participants [9], and we postulated that this change may be indicative of the glyoxylate shunt, which is activated during oxidative stress to provide an alternate metabolic route to the citric acid cycle [45]. Oxidative stress following TBI may initiate this metabolic pathway. Interestingly, the citrate or TCA cycle itself was also found to be significantly altered, as evidenced by a significant change in the concentration of the pathway intermediates citrate and pyruvate in the participants’ serum. A similar disruption was also seen in the serum of our SCI study discussed above [9]. This alteration suggests a metabolic switch from aerobic respiration to anaerobic respiration due to ischemia following injury [46]. This shift in metabolic mode may also indicate secondary tissue damage following TBI.

Finally, TBI potentially altered the glycine, serine, and threonine metabolic pathway. Evidence suggests that plasma amino acids tend to be higher in individuals with skeletal muscle degeneration due to higher protein requirements [47]. In a previous study using a mouse model, it was shown that TBI induced atrophy in the lower limb muscles, such as the soleus and tibialis anterior [48]. Thus, changes in body composition following brain trauma, likely due to immobility from paresis, may underlie these inferred changes in blood amino acid levels.

The single metabolite that showed an association with the GCS was L-alanine, demonstrating a negative correlation. This observation indicates that an increase in serum L-alanine levels is associated with poorer GCS scores. L-alanine is a non-essential amino acid and is formed from pyruvate via the enzyme alanine transaminase and is then shuttled to the liver where its carbon skeleton is converted to glucose [49]. Ischemia following TBI may provoke increased demand for glucose and, therefore, conversion from its precursors, such as L-alanine. Furthermore, the ratio of alanine to glutamate has been shown to serve as an index to predict tissue survival following cerebral ischemia, whereby a decrease in this ratio predicts a less severe injury in a gerbil model [50]. Potentially, a similar mechanism exists within the human brain. Hence, more research on L-alanine and its involvement in TBI severity is warranted.

### 4.2. Limitations

Although the sample size in the present study was limited, the within-subject design ensured that the regulation of metabolite concentrations provided a more robust indicator of change. Further validation will be needed to ascertain the prognostic potential of the identified metabolites in clinical practice. Given that the present investigation focused only on males, a more diverse cohort including females and individuals of diverse ethnic backgrounds is needed. Future studies should also consider the potential confounds of diet, exercise, body mass index, medical history, and acute versus chronic drug treatment when developing the study design. To account for injury in general, similar studies would also benefit from a musculoskeletal injury control group. Nevertheless, these preliminary findings indicate that specific metabolic profiles can be associated with symptom severity. Thus, this exploratory pilot study provides substantiated evidence for the potential of metabolites in clinically available biofluids for prognosticating outcomes in males following TBI.

## 5. Conclusions

The goal of the present exploratory pilot prospective cohort study was to create biomarker profiles indicative of clinical severity in TBI by detecting variances in metabolomic profiles across two time points, unveiling biochemical pathways derived from significant metabolites and exploring connections between metabolic alterations and the severity of the injury. While striving to meet these aims, we identified endogenous blood-derived metabolites that changed throughout recovery following TBI and identified eight potentially associated biochemical pathways. We also found that L-alanine was significantly negatively correlated with the severity of TBI injury (GCS), suggesting it may be a metabolite to explore in further studies. Although the sample size in the present study was small, the within-subject design ensured the regulation of metabolite concentrations over time. Metabolite profiling and pathway analysis should be further explored in subsequent studies related to the measurement of injury severity in participants with TBI. Further validation will ascertain the potential of the identified metabolites as proxy measures for clinical use. By unveiling pathological processes in the brain, a metabolomics perspective has the potential to complement standard clinical outcome measures and improve clinical decision-making.

## Figures and Tables

**Table 1:** Participant characteristics (*n* = 8 males) indicating age, TBI type, initial Glasgow Coma Scale score, co-morbidities, medication use, days between TBI and blood and clinical assessment collection at baseline and 6 months, and both the initial and 6 months post-injury Montreal Cognitive Assessment (MoCA) and Functional Independence Measure (FIM) scores.

Participant Code	TBI Type	Glasgow Coma Scale Score	Age	Co-Morbidities	Medications	Blood Collection (Days Post-Injury)		Clinical Assessments (Days Post-Injury)		MoCA		FIM	
						Initial	6 Month	Initial	6 Month	Initial	6 Month	Initial	6 Month
**TBI_02**	Frontal	3	18	Injury to right ear, right fracture petrous temporal bone	Tylenol	3	218	4	218	25	30	126	126
**TBI_03**	Frontal	10	49	Depression, asthma, EtOH abuse	Docusate sodium, fentanyl, lorazepam, phenytoin, senokot, thiomine, tobradex, multi-vits	1	312	20	312	23	26	113	122
**TBI_07**	SDH	6	18	None	None	4	226	59	226	26	27	124	125
**TBI_13**	DAI-Left	13	64	Multiple face lacerations, nasal fracture, liver laceration, dental injuries	Acetaminophen, docusate sodium, heparin, quetiapine	4	200	33	200	20	27	112	121
**TBI_19**	SDH/SAH Bifrontal	8	46	None	Trazadone, testosterone, seroquel	2	197	31	197	25	26	113	124
**TBI_24**	SDH/SAH	15	68	Chronic lower back pain, liver laceration, bilateral shoulder injuries, torn right rotator cuff	None	3	198	70	198	21	23	126	123
**TBI_26**	SDH/SAH	14	48	None	Tylenol	2	184	29	184	27	27	124	126
**TBI_29**	SAH-Right Frontal	12	48	L2, L4, L5 fracture, sciatic nerve damage, eczema, history of smoking	Tylenol, baclofen, panoloc	2	NaN	16	NaN	23	23	115	122

Abbreviations: SDH = subdural hematoma, SAH = subarachnoid hemorrhage, DAI = diffuse axonal injury.

**Table 2:** Metabolites displaying statistical significance for *n* = 8 male participants with TBI, according to the paired *t*-test/Wilcoxon–Mann–Whitney tests and VIAVC analysis. Blood-derived metabolites are displayed in order of significance (*p* < 0.05) based on the paired *t*-test/Wilcoxon–Mann–Whitney test. Significance by the VIAVC F-ranked subset is indicated by a single dagger (**†**), and significance by the VIAVC best subset is indicated by the double dagger (**††**). Each metabolite’s corresponding chemical shift and regulation with the percent difference is displayed. The metabolites with multiple significant resonance peaks are reported as Metabolite.1, Metabolite.2, … Metabolite.n.

Metabolite	Chemical Shift (ppm)	Paired t/Wilcoxon *p*-Value	Regulation (% Difference)
**L-Phenylalanine.1**	7.362	0.0002	Down (−60.140%)
**1,9-Dimethyluric Acid**	3.290	0.0005	Up (25.651%)
**Phosphonoacetate.1**	2.657	0.0012	Up (42.262%)
**p-Cresol.1**	7.159	0.0013	Down (−84.436%)
**L-Phenylalanine.2**	7.379	0.0031	Down (−30.914%)
**Glycine.1**	3.582	0.0031	Up (18.260%)
**Citric Acid**	2.536	0.0040	Up (35.800%)
**Phosphonoacetate.2**	2.672	0.0045	Up (37.428%)
**1,3-Dimethyluric Acid.1**	3.295	0.0046	Up (22.159%)
**p-Cresol.2**	7.144	0.0050	Down (−65.625%)
**2-Hydroxybutyrate ††**	0.904	0.0050	Down (−79.804%)
**Glycine.2**	3.567	0.0061	Up (19.568%)
**Trimethylamine-N-Oxide**	3.285	0.0066	Up (26.092%)
**3-Methyl-2-Oxovaleric Acid**	0.915	0.0066	Down (−75.379%)
**Creatinine.1**	3.054	0.0078 (W)	Up (21.179%)
**Levulinate †**	2.456	0.0089	Up (20.412%)
**Unidentified Multiplet**	0.980	0.0107	Down (−53.026%)
**Citramalic Acid.1**	2.478	0.0112	Up (18.255%)
**4-Pyridoxate**	2.445	0.0153	Up (16.975%)
**1,5-Anhydrosorbitol.1**	3.360	0.0156 (W)	Up (27.891%)
**1,3-Dimethyluric Acid.2 †**	3.300	0.0156 (W)	Up (20.480%)
**Citramalic Acid.2 †**	2.489	0.0158	Up (17.716%)
**Pyruvic Acid**	2.467	0.0169	Up (17.951%)
**L-Alanine ††**	1.493	0.0172	Up (35.621%)
**1,5-Anhydrosorbitol.2**	3.280	0.0178	Up (20.012%)
**Guanidoacetate †**	3.804	0.0209	Up (15.256%)
**5-Hydroxyindole-3-acetate**	3.572	0.0221	Up (20.959%)
**Tyrosine**	6.930	0.0234	Down (−33.767%)
**Glucose.1**	3.461	0.0274	Down (−14.882%)
**Glucose.2**	3.821	0.0322	Down (−9.187%)
**Unidentified Singlet**	1.212	0.0352	Down (−86.139%)
**D-Mannose**	5.196	0.0391 (W)	Down (−76.053%)
**Hydroxyphenylacetylglycine**	3.600	0.0391 (W)	Up (11.043%)
**Theophylline**	3.564	0.0421	Up (14.543%)
**Lactate.1**	4.109	0.0428	Up (25.843%)
**Glucose.3**	3.856	0.0438	Down (−7.688%)
**π-methylhistidine**	7.970	0.0449	Up (18.003%)
**Unidentified Multiplet**	2.433	0.0476	Up (15.001%)
**Creatinine.2**	4.065	0.0487	Up (24.941%)
**Acetylphosphate**	2.132	0.0490	Up (14.059%)
**Methylsuccinic Acid**	2.149	0.0497	Up (13.903%)
**Lactate.2 †**	4.135	0.0571	Up (29.606%)
**1,3,7-Trimethyluric Acid ††**	3.337	0.0799	Up (21.040%)
**α-Ketoisovaleric Acid †**	1.131	0.1389	Down (−30.615%)
**Pantothenic Acid †**	0.943	0.2278	Down (−26.969%)

## Data Availability

The data presented in this study have not been uploaded to an online database accessible to the public due to privacy restrictions. However, the corresponding authors will make the data available upon request.
